# Recent Developments in Vaccines for Viral Diseases

**DOI:** 10.3390/vaccines11020198

**Published:** 2023-01-17

**Authors:** Yasir Waheed, Ranjit Sah, Khalid Muhammad

**Affiliations:** 1 Office of Research, Innovation and Commercialization (ORIC), Shaheed Zulfiqar Ali Bhutto Medical University (SZABMU), Islamabad 44000, Pakistan; 2 Gilbert and Rose-Marie Chagoury School of Medicine, Lebanese American University, Byblos 1401, Lebanon; 3 Department of Microbiology, Institute of Medicine, Tribhuvan University Teaching Hospital, Kathmandu 44600, Nepal; 4 Department of Microbiology, Dr. D. Y. Patil Medical College, Hospital and Research Center, Dr. D. Y. Patil Vidyapeeth, Pune 411018, India; 5 Department of Biology, College of Science, UAE University, Al Ain 15551, United Arab Emirates

The world is continuously facing the threat of emerging infectious diseases. The COVID-19 pandemic has affected over 200 countries and territories of the world, and has also severely affected the world’s healthcare and financial systems [1]. In 2022, Mpox (Monkeypox) cases were reported from all six WHO regions [2]. Additionally, Ebola cases are still being reported from Africa. Therefore, it is crucial to speed up the vaccine development efforts against different viral diseases, and make the vaccine reachable to as many people in the world as possible, irrespective of their power to purchase it. This article highlights the vaccine development progress made for the major viral diseases. 

The current COVID-19 pandemic has caused more than 661 million infections with over 6.6 million deaths worldwide [1]. The first COVID-19 case was reported in December 2019, and the first genome sequence of COVID-19 was published in January 2020. The global scientific community thus commenced efforts to make a vaccine for COVID-19, and the first COVID-19 vaccine was authorized in mid-2020. By the end of 2020, many COVID-19 vaccines were authorized. As of 25 December 2022, 68.7% of the world population has received at least one dose of a COVID-19 vaccine, and a total of 13.09 billion COVID-19 vaccine doses have been administered globally [3]. Approximately, 14.4 million to 19.8 million deaths are prevented due to COVID-19 vaccination in a year [4]. 

Approximately, 38.4 million people are living with HIV worldwide [5]. In the last 20 years, some success in the fight against HIV has been achieved, with the availability of anti-viral therapy in endemic regions. However, a total cure has not been found, and an effective vaccine has not yet been designed to eradicate HIV from the world. Different approaches have been used to design a vaccine for HIV, but only a few of them show some efficacy. Unfortunately, these vaccines only limit the viral infection to a certain degree, and they fail to improve CD4 T-cell count, reduce viral load, and cause a delay in HIV disease progression [6]. 

Dengue virus is the causative agent of dengue fever, and multiple other health complications. Approximately 100–400 million dengue cases occur annually, of which 80% remain mild and asymptomatic, while 20% cause severe infection, leading to 22,000 deaths annually [7]. The US FDA approved Dengvaxia vaccine is available for use in children and adolescents 9–16 years old, with laboratory confirmed evidence of previous dengue virus, and living in dengue endemic areas. Dengvaxia, however, causes a more severe dengue infection if administered to a person who has never had a dengue infection [8]. Takeda’s dengue vaccine (TAK-003) is on the FDA list for approval. TAK-003 can prevent dengue disease from any of the four serotypes of the virus in individuals aged 4–60 years. Takeda’s vaccine showed 80% efficacy for preventing symptomatic infection at 12 months, and 90% efficacy for preventing hospitalization at 18 months [9]. 

The Zika virus is an emerging infectious disease of great public health importance. Most of the infections caused by Zika virus remain asymptomatic or mildly symptomatic. While some Zika infections are associated with microcephaly in children born from mothers infected with Zika during pregnancy, in adults, Zika infection is responsible for Guillain Barre Syndrome. Several Zika virus vaccines are at different stages of development, but none of the vaccines have been approved by the FDA [10]. 

In 2022, more than 83,000 cases of Mpox (Monkeypox) have been reported from 110 countries around the world [2]. Previous Mpox cases were reported in a few African countries, but this time, the Mpox cases have been reported in all six WHO regions. With the exception of a few African countries, the current outbreak of Mpox mainly affects men who have sex with men, and who have had recent sexual interaction with one or multiple partners. The five most affected countries by the current Mpox outbreak are the USA, Brazil, Spain, France, and Colombia. JYNNEOS is an FDA approved vaccine for Mpox, and it was developed to protect against both Mpox and smallpox. The vaccine can be administered to both children and adults who are at risk of contracting Mpox. JYNNEOS is a two-dose vaccine; the second dose should be given 4 weeks after the first dose, and the maximum level of protection is expected after 14 days of receiving the second dose of the JYNNEOS vaccine [11].

Human papillomavirus (HPV) is one of the most potent viruses, and it causes sexually transmitted diseases on a large scale worldwide. Approximately, over 150 types of human papillomavirus have been identified, and nearly 40 of them lead to infectious cycles in the host [12]. Many HPV infections cause no symptoms and resolve in two years, however some of them lead to warts or precancerous lesions. HPV16 and HPV18 account for 70% of cervical cancers, whereas HPV6 and HPV11 are the major causative agents of genital warts and laryngeal papillomatosis. Three HPV vaccines are approved by the FDA: Gardasil (quadrivalent vaccine), Cervarix (bivalent vaccine), and Gardasil 9 (nanovalent vaccine). In the last few years, Gardasil 9 has been the most widely used vaccine for HPV; it provides protection against nine HPV types, namely HPV6, HPV11, HPV16, HPV18, HPV31, HPV33, HPV45, HPV52, and HPV58 [13]. 

Polio is a highly contagious virus that is responsible for causing disability and life threating disease. Due to unified global polio eradication efforts, there has been a 99.9% reduction in global polio cases. Polio is endemic in only two countries, but recently some single polio outbreaks have been reported in high-income countries [14]. It is important to eliminate the misconceptions regarding polio vaccinations, and to minimize the number of children who miss polio vaccination in endemic countries, in order to achieve global polio eradication [15].

Globally, 1.1 million people are dying each year from viral hepatitis. The World Health Organization has developed a strategy to eliminate hepatitis by 2030. Luckily, a highly effective vaccine is available for the Hepatitis B virus. The coverage of the Hepatitis B virus vaccine is 90% among children, but there is a need to increase the birth dose of HBV vaccination in newborns to prevent the vertical transmission of the Hepatitis B virus [16]. There is no vaccine available for the Hepatitis C virus. Only a virus-like particle-based vaccine candidate made it into phase 2 clinical trials; however, the vaccine failed to provide protection from HCV infection [17]. 

There have been several outbreaks of the Ebola virus in the last ten years. Several vaccine development techniques, including inactivated vaccines, DNA base vaccines, virus-like particles, virus-like replicon particles, recombinant viral vector vaccines, and plant-based vaccines were used to develop vaccines for the Ebola virus [18]. The only FDA approved Ebola vaccine for use in individuals 18 years or older is ERVEBO. The vaccine was administered to over 35,000 individuals in the country of Guinea, and to individuals in Congo during the 2018–2020 Ebola outbreak. ERVEBO showed good safety and effectiveness against the Zaire Ebola virus [19]. 

Ultimately, viruses are continuously evolving and some of them have high mutation rates. Multiple variants of COVID-19 have shown different disease transmission and infectivity patterns [20,21]. The COVID-19 pandemic has united the world in the efforts to develop a vaccine, and within a period of six months, the first COVID-19 vaccine received authorization. Currently, there is a need to use the latest advancements in science to develop a better vaccine. Many research groups are working on the universal vaccine concept, in order to develop a broader range of vaccines which can protect from multiple variants of a virus. Unified efforts are required to make the vaccines available to the whole world, and especially to those countries who cannot afford vaccines. 

## References

[1] Worldometer Coronavirus Cases. https://www.worldometers.info/coronavirus/.

[2] World Health Organization (2022). 2022 Mpox (Monkeypox) Outbreak: Global Trends. https://worldhealthorg.shinyapps.io/mpx_global/#section-fns2‘2022%20Monkeypox%20Outbreak:%20Global%20Trends’.

[3] Our World in Data Coronavirus (COVID-19) Vaccination. https://ourworldindata.org/covid-vaccinations.

[4] Watson J.O., Barnsley G., Toor J., Hogan A.B., Winskill P., Ghani A.C. (2022). Global Impact of the first year of COVID-19 vaccination: A mathematical modelling study. Lancet Infect. Dis..

[5] (2022). UNAIDS. https://www.unaids.org/en/resources/fact-sheet#:~:text=38.4%20million%20%5B33.9%20million%E2%80%9343.8,accessing%20antiretroviral%20therapy%20in%202021.

[6] Collaboration H.C. (2010). The effect of combined antiretroviral therapy on the overall mortality of HIV-infected individuals. AIDS.

[7] World Health Organization (2022). Fact Sheet Dengue and Severe Dengue. https://www.who.int/news-room/fact-sheets/detail/dengue-and-severe-dengue.

[8] Thomas S.J., Yoon I.K. (2019). A review of Dengvaxia®: Development to deployment. Hum. Vaccines Immunother..

[9] López-Medina E., Biswal S., Saez-Llorens X., Borja-Tabora C., Bravo L., Sirivichayakul C., Vargas L.M., Alera M.T., Velásquez H., Reynales H. (2022). Efficacy of a Dengue Vaccine Candidate (TAK-003) in Healthy Children and Adolescents 2 Years after Vaccination. J. Infect. Dis..

[10] Jamil Z., Waheed Y., Durrani T.Z. (2016). Zika virus, a pathway to new challenges. APJTM.

[11] Centers for Disease Control and Prevention (2022). Mpox Vaccination Basics. https://www.cdc.gov/poxvirus/monkeypox/vaccines/vaccine-basics.html.

[12] (2022). NIH; National Cancer Institute. https://www.cancer.gov/about-cancer/causes-prevention/risk/infectious-agents/hpv-vaccine-fact-sheet#:~:text=Three%20vaccines%20that%20prevent%20infection,%2C%20Gardasil%209%2C%20and%20Cervarix.

[13] Malik S., Sah R., Muhammad K., Waheed Y. (2023). Tracking HPV infection, associated cancer development and recent treatment efforts—A comprehensive review. Vaccines.

[14] Malik S., Waheed Y. (2022). Tracking down the recent surge of polio virus in endemic and outbreak countries. J. Med. Virol..

[15] Waheed Y. (2018). Polio eradication challenges in Pakistan. Clin. Microbiol. Infect..

[16] Waheed Y. (2021). Progress on global hepatitis elimination targets. World J. Gastroenterol..

[17] Page K., Melia M.T., Veenhuis R.T., Winter M., Rousseau K.E., Massaccesi G., Osburn W.O., Forman M., Thomas E., Thornton K. (2021). Randomized Trial of a vaccine regimen to prevent chronic HCV infection. N. Engl. J. Med..

[18] World Health Organization Fact Sheets Ebola Virus Diseases. https://www.who.int/news-room/fact-sheets/detail/ebola-virus-disease?gclid=Cj0KCQiA-oqdBhDfARIsAO0TrGG71Xt38TR7yRQTtdzlbnchs2bkNL-Svl53Ecm757FolVhTVx0iTloaAkr8EALw_wcB.

[19] Woolsey C., Geisbert T.W. (2021). Current state of Ebola virus vaccines: A snapshot. PLoS Pathog..

[20] Khan A., Waris H., Rafique M., Suleman M., Mohammad A., Ali S.S., Khan T., Waheed Y., Liao C., Wei D.-Q. (2022). The Omicron (B.1.1.529) variant of SARS-CoV-2 binds to the hACE2 receptor more strongly and escapes the antibody response: Insights from structural and simulation data. Int. J. Biol. Macromol..

[21] Wang J., Muhammad S.F., Aman S., Khan A., Munir S., Khan M., Mohammad A., Waheedi Y., Munir M., Guo L. (2022). Strucutural communication fingerprinting and dynamic investigation of RBD-hACE2 complex from BA.1 x AY.4 recombinant variant (Deltacron) of SARS-CoV-2 to decipher the structural basis for an enhaced transmission. J. Biomol. Struct. Dyn..

